# CNBP Homologues Gis2 and Znf9 Interact with a Putative G-Quadruplex-Forming 3′ Untranslated Region, Altering Polysome Association and Stress Tolerance in *Cryptococcus neoformans*

**DOI:** 10.1128/mSphere.00201-18

**Published:** 2018-08-08

**Authors:** Jay Leipheimer, Amanda L. M. Bloom, Tilman Baumstark, John C. Panepinto

**Affiliations:** a Department of Microbiology and Immunology, Witebsky Center for Microbial Pathogenesis and Immunology, Jacobs School of Medicine and Biomedical Sciences, University at Buffalo, Buffalo, New York, USA; Yonsei University

**Keywords:** CNBP, G-quadruplex, posttranscriptional gene regulation, stress response, translation

## Abstract

In Cryptococcus neoformans, mRNAs encoding ribosomal proteins (RP) are rapidly and specifically repressed during cellular stress, and the bulk of this repression is mediated by deadenylation-dependent mRNA decay. A motif-finding approach was applied to the 3′ untranslated regions (UTRs) of RP transcripts regulated by mRNA decay, and a single, significant motif, GGAUG, was identified. Znf9, a small zinc knuckle RNA binding protein identified by mass spectrometry, was found to interact specifically with the *RPL2* 3′-UTR probe. A second, homologous protein, Gis2, was identified in the genome of C. neoformans and also bound the 3′-UTR probe, and deletion of both genes resulted in loss of binding in cell extracts. The *RPL2* 3′ UTR contains four G-triplets (GGG) that have the potential to form a G-quadruplex, and temperature gradient gel electrophoresis revealed a potassium-dependent structure consistent with a G-quadruplex that was abrogated by mutation of G-triplets. However, deletion of G-triplets did not abrogate the binding of either Znf9 or Gis2, suggesting that these proteins either bind irrespective of structure or act to prevent structure formation. Deletion of both *GIS2* and *ZNF9* resulted in a modest increase in basal stability of the *RPL2* mRNA which resulted in an association with higher-molecular-weight polysomes under unstressed conditions. The *gis2*Δ mutant and *gis2*Δ *znf9*Δ double mutant exhibited sensitivity to cobalt chloride, fluconazole, and oxidative stress, and although transcriptional induction of *ERG25* was similar to that of the wild type, analysis of sterol content revealed repressed levels of sterols in the *gis2*Δ and *gis2*Δ *znf9*Δ double mutant, suggesting a role in translational regulation of sterol biosynthesis.

**IMPORTANCE** Stress adaptation is fundamental to the success of Cryptococcus neoformans as a human pathogen and requires a reprogramming of the translating pool of mRNA. This reprogramming begins with the regulated degradation of mRNAs encoding the translational machinery. The mechanism by which these mRNAs are specified has not been determined. This study has identified a *cis* element within a G-quadruplex structure that binds two C. neoformans homologues of cellular nucleic acid binding protein (CNBP). These proteins regulate the polysome association of the target mRNA but perform functions related to sterol homeostasis which appear independent of ribosomal protein mRNAs. The presence of two CNBP homologues in C. neoformans suggests a diversification of function of these proteins, one of which appears to regulate sterol biosynthesis and fluconazole sensitivity.

## Abstract (precis)

Stress adaptation is fundamental to the success of Cryptococcus neoformans as a human pathogen and requires a reprogramming of the translating pool of mRNA. This reprogramming begins with the regulated degradation of mRNAs encoding the translational machinery. The mechanism by which these mRNAs are specified has not been determined. This study has identified a *cis* element within a G-quadruplex structure that binds two C. neoformans homologues of cellular nucleic acid binding protein (CNBP). These proteins regulate the polysome association of the target mRNA but perform functions related to sterol homeostasis which appear independent of ribosomal protein mRNAs. The presence of two CNBP homologues in C. neoformans suggests a diversification of function of these proteins, one of which appears to regulate sterol biosynthesis and fluconazole sensitivity.

## INTRODUCTION

The fungal pathogen Cryptococcus neoformans employs posttranscriptional regulation of gene expression as part of the transcriptome reprogramming that accompanies cellular stress (1–3). This complex adaptive reprogramming is an important part of pathogenesis and includes the rapid degradation of mRNAs encoding the components of translational machinery. The stress-induced degradation of ribosomal protein (RP) mRNAs is mediated by the major cytoplasmic deadenylase, Ccr4. However, the mechanism by which these mRNAs are specified for degradation is yet unknown.

Often, *cis* elements in the 3′ untranslated regions (UTRs) of mRNAs encode proteins with roles in the fates of the mRNAs, including stability, translatability, and localization. These aspects of mRNA fate can be regulated in *cis* by structural elements or in *trans* through the recognition of *cis* elements by RNA binding proteins. G-quadruplexes are an example of structural elements that can control mRNA fate (4–7). G-quadruplexes are formed through a combination of Watson-Crick and Hoogstein base pairing in which four guanosine residues coordinate a potassium ion and stack in combinations of two or three quadruplexes (8, 9). These structures, which can occur in both DNA and RNA, can impede processivity of telomerase or impair translation (10–12).

Eukaryotes have evolved an RNA binding protein purported to prevent the occurrence of G-quadruplex formation (13, 14). In mammals, cellular nucleic acid binding protein (CNBP) interacts with G-rich sequences and promotes translation of putative G-quadruplex-containing mRNAs. CNBP is essential in mammals, with mutations resulting in embryonic lethality in mice (15). Interestingly, nucleotide repeat expansions in the first intron of CNBP are implicated in the development of myotonic dystrophy type 2 (16). The role of CNBP orthologues in lower eukaryotes is less clear.

In this study, we performed an open-ended identification of putative elements that could mediate the posttranscriptional regulation of RP transcripts in C. neoformans. This analysis revealed a G-rich sequence within the context of a putative G-quadruplex and two orthologues of mammalian CNBP that bind it. Characterization of the RNA structure of 50-base RNA constructs comprised of this region revealed adoption of a potassium ion-dependent conformation *in vitro*, consistent with a G-quadruplex-containing structure. Deletion of *GIS2* and *ZNF9* revealed a role for these proteins in regulation of RP transcripts under unstressed conditions and RP transcript-independent functions of these two CNBP orthologues in susceptibility to fluconazole, cobalt chloride, and peroxide stress.

## RESULTS

### Gis2 and Znf9 interact with a 3′-UTR element in RP transcript 3′ UTRs.

**FIG 1:**
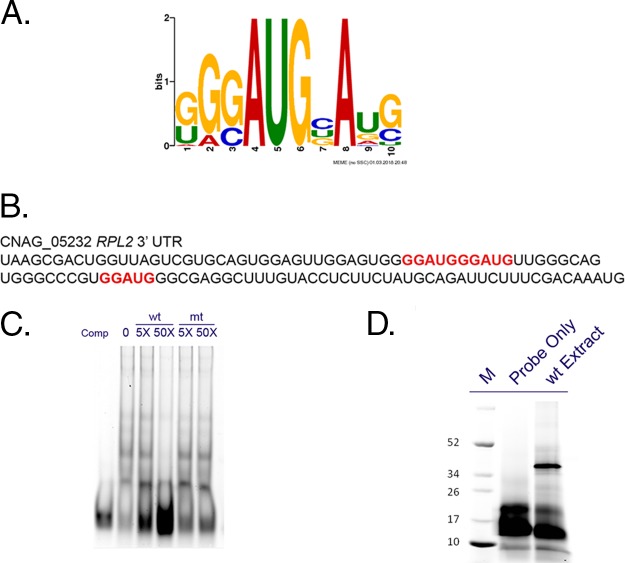
The 3′ UTRs of ribosomal protein mRNAs contain a GGAUG element that binds protein with specificity. (A) MEME analysis revealed a single significant element with an invariant AUG codon in a G-rich context that we are referring to as the GGAUG element. (B) The sequence of the RNA oligonucleotide used in the EMSA reactions with the GGAUG elements highlighted in red. (C) Native EMSA from C. neoformans cell extracts using the same TYE705-labeled RNA oligonucleotide in the presence of either 5× or 50× molar excess of an unlabeled competitor (wild type [wt]) or competitor (Comp) in which the element was mutated (mt). (D) Cross-linked EMSA demonstrating the protein binding activity of a TYE705-labeled RNA oligonucleotide encompassing the GGAUG element from *RPL2* with cell extract of wild-type (wt) C. neoformans. The positions (in kilodaltons) of molecular mass markers (M) are shown to the left of the gel.

RP transcripts are coregulated, and in response to cellular stress, they are rapidly repressed through transcriptional repression and accelerated mRNA degradation. In the C. neoformans fungal pathogen, deadenylation-dependent mRNA decay is required for the accelerated degradation of RP transcripts and deletion of the major mRNA deadenylase, Ccr4, results in stabilization of these mRNAs (1, 2, 17). The features of RP transcripts that confer specificity to stress-responsive degradation are unknown, and so we employed a bioinformatic tool, MEME, to identify conserved sequences in the 3′ UTRs of RP transcripts that might confer this specificity (18, 19). The sequence set used for motif discovery was the 3′-UTR sequences of 35 RP transcripts that were found to be significantly upregulated in the *ccr4*Δ mutant 10 min after a shift to 37°C (17). A single, significant motif was discovered that contained a conserved core GGAUG element flanked by G- and U-rich sequences (Fig. 1A). To determine whether this sequence exhibited specific protein-binding capacity, we generated a 50-base RNA oligonucleotide consisting of the sequence harboring this element from the *RLP2* mRNA (Fig. 1B and Table 1), which contained a direct repeat of the GGAUG element and flanking sequence both up- and downstream of the core sequence. The oligonucleotide was synthesized with a TYE705 infrared fluorescence label for use in electrophoretic mobility shift assays. Incubation of the oligonucleotide with cell extracts of C. neoformans resulted in a shift that was competed by the addition of 5 and 50 M excess of unlabeled oligonucleotide (Fig. 1C). A mutant competitor, in which the GGAUG element was mutated to AACCA, was unable to compete for binding to the labeled oligonucleotide, suggesting that the interaction is specific to the element or structure conferred by the GGAUG sequence. To estimate the size of the interacting protein, we used UV cross-linking followed by SDS-PAGE to resolve the protein-oligonucleotide complex (Fig. 1D). A single band was detected in the 40-kDa range. This suggested that the interacting protein was approximately 20 kDa in molecular weight, given that the labeled RNA oligonucleotide ran at approximately 20 kDa in the absence of protein.

**TABLE 1:** DNA and RNA oligonucleotides used in this study

Oligonucleotide name	Oligonucleotide sequence^a^
DNA oligonucleotides	
F-rZNF9-BglII	TAATAAAGATCTGATGTTTGGAGCTGCTGCTGTTCC
R-rZNF9-BglII	TAATAAAGATCTCAAGCACAGATACTATTACTCCGC
F-ZNF9upKO-XbaI	TAATAATCTAGAAGTAAGATCTTCTGCCCAGGCG
R-ZNF9upKO-BglII	TAATAAAGATCTGCCGTGTTCCTTCGTTGG
F-ZNF9downKO-MunI	TAATAACAATTGCATGACTCATCACTGACTGC
R-ZNF9downKO-XhoI	TAATAACTCGAGCAGATAAAGTGCTGAAGAGGC
F-NAT-BglII	TAATAAAGATCTGCTGCGAGGATGTGAGCTGG
R-NAT-MunI	TAATAACAATTGAAGCTTATAGAAGAGATGTAGAAACTAGC
F-GIS2upKO-XbaI	TAATAATCTAGAGGGCATCAACAAAGTTTGC
R-GIS2upKO-BglII	TAATAAAGATCTCTCAGAAAGCAAGTGGGTGG
F-GIS2downKO-MunI	TAATAACAATTGTCGTTGTTGGATTGTAAGCG
R-GIS2downKO-XhoI	TAATAACTCGAGGAGAACAGCAAGAGCGACG
F-NEO-BglII	TAATAAAGATCTCAGGATTCGAGTGGCATGG
R-NEO-BglII	TAATAACAATTGCGACGGCCAGTGAATTGTAATACG
F-GIS2cDNA-BamHI	TAATAAGGATCCGATGTTCGGTGCTCCTCGAGG
R-GIS2cDNA-BamHI	TAATAAGGATCCTTAGGCAGCAGGGGCTTCAGC
F-GIS2complement	TGCAGGATGAGGAGACAGC
R-GIS2complement	GATGACCACGGTGTGATCG
F-ERG25-probe	TCGACAAGTACATCCCCGG
R-ERG25-probe	CGTTCTTTCCCCGCTTGCC

RNA oligonucleotides	
TYE705-RPL2-3′UTR	5′-TYE705-UGCAGUGGAGUUGGAGUGGGGAUGGGAUGUUGGGCAGUGGGCCCGUGGAU
Unlabeled competitor	UGCAGUGGAGUUGGAGUGGGGAUGGGAUGUUGGGCAGUGGGCCCGUGGAU
mt competitor	UGCAGUGGAGUUGGAGUGAAACCAAACCGUUGGGCAGUGGGCCCGUGGAU
Biotin-RPL2-3′UTR	5′-biotin-UGCAGUGGAGUUGGAGUGGGGAUGGGAUGUUGGGCAGUGGGCCCGUGGAU
Biotin-RPL2-3′UTRmt	5′-biotin-UGCAGUGGAGUUGGAGUGAAACCAAACCGUUGGGCAGUGGGCCCGUGGAU

a The mutation introduced into the RNA oligonucleotide is shown underlined.

**FIG 2:**
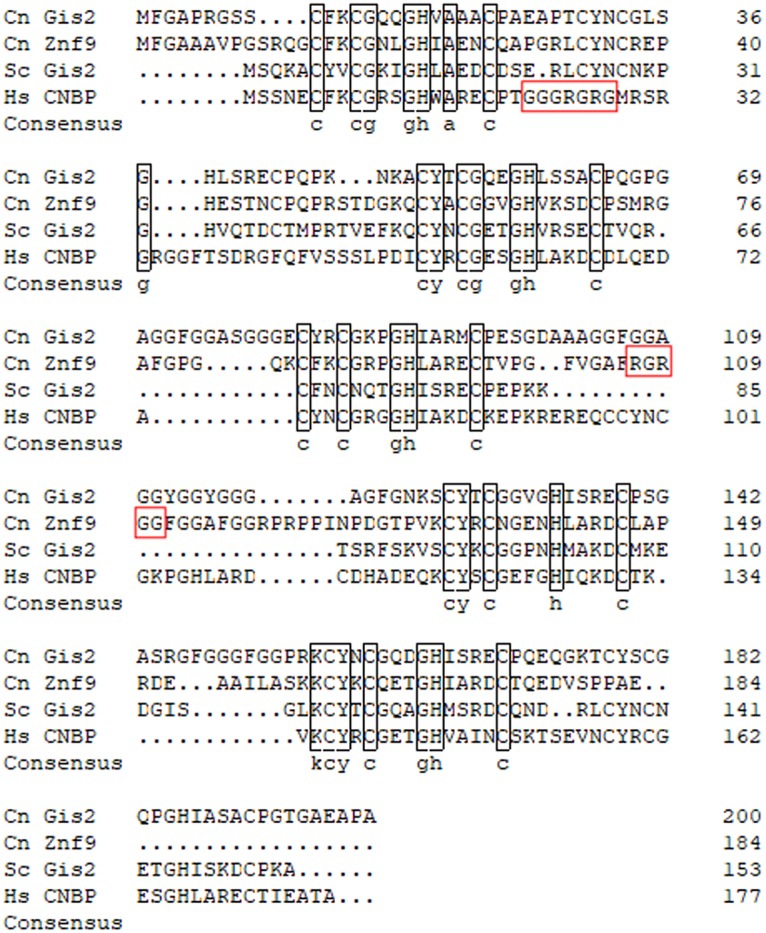
Protein sequence alignment of Gis2 and Znf9 with the S. cerevisiae and human orthologues. Protein sequences of Gis2 (CNAG_02338) and Znf9 (CNAG_01273) were aligned with sequences of S. cerevisiae Gis2p (KZV08368.1) and human CNBP (P62633.1) using DNAMAN software with the Gonnet protein weight matrix. Black boxes indicate identity within the zinc knuckle domains, and red boxes indicate consensus arginine methylation motifs. Cn, C. neoformans; Sc, S. cerevisiae; Hs, *Homo sapiens*.

We went on to identify the interacting protein by affinity chromatography. The same RNA oligonucleotide used in the electrophoretic mobility shift assays (EMSAs) was synthesized with a biotin label and used to pull down putative interacting proteins. The region of the gel lane from the pulldown corresponding to ~20 kDa, as well as the corresponding region from a pulldown performed in parallel with a biotinylated version of the mutant competitor oligonucleotide was submitted for liquid chromatography coupled to tandem mass spectrometry (LC-MS/MS) analysis. Within the list of proteins uniquely identified in the experimental pulldown, but absent in the control, was a zinc knuckle RNA binding protein of ~20 kDa with homology to human cellular nucleic acid binding protein (CNBP/Znf9) and Gis2 from Saccharomyces cerevisiae. A subsequent reannotation of the C. neoformans genome revealed a second CNBP homologue. Alignment of the protein sequence of these two CNBP homologues with that of S. cerevisiae Gis2p and human CNBP revealed high similarity within the zinc knuckle domains (Fig. 2). A distinguishing feature is the presence of an RG-box in Znf9 which is a common target for posttranslational modification by protein arginine methylation (20). The human CNBP/Znf9 also contains an RG-box that is subject to arginine methylation (21). Because of this feature, we have named the cryptococcal protein containing the RG-box Znf9, and we have named the second homologue for the S. cerevisiae homologue, Gis2.

**FIG 3:**
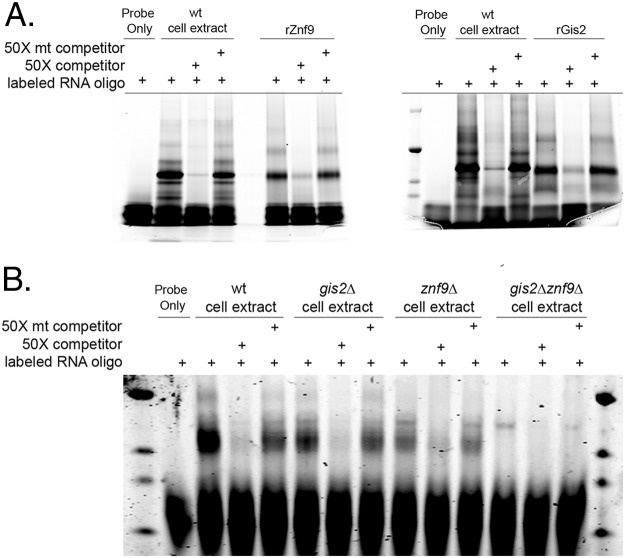
Both Gis2 and Znf9 bind the GGAUG element with specificity. (A) UV-cross-linked EMSA with either cell extract or recombinant Znf9 (rZnf9) (left) or recombinant Gis2 (rGis2) (right) with the TYE-705 RNA oligonucleotide alone or in the presence of 50× wild type or 50× mutant (mt) unlabeled competitor oligonucleotide. (B) UV cross-linked EMSA analysis of cell extracts of the wild type, *gis2*Δ mutant, *znf9*Δ mutant, and *gis2*Δ *znf9*Δ double mutant with the TYE705-labeled RNA oligonucleotide alone or in the presence of 50× wild type or 50× mutant unlabeled competitor.

To validate the ability of these proteins to bind the identified motif with specificity, both proteins were produced recombinantly in Escherichia coli and assessed for binding to the *RPL2* RNA oligonucleotide by UV-cross-linked EMSA. As demonstrated in Fig. 3A, both proteins bound the *RPL2* oligonucleotide with specificity. To determine whether both proteins in cellular extracts were able to bind the identified element, we generated single deletion mutants of each locus and a double deletion mutant in the H99 background. In cell extracts of the *gis2*Δ or *znf9*Δ single mutants, EMSAs revealed the maintenance of the interacting band that was competed with the unlabeled competitor (Fig. 3B). Only in EMSAs using extracts from the *gis2*Δ *znf9*Δ double mutant was the interacting band absent, suggesting that both proteins are able to interact with the GGAUG element *in vivo*.

### The GGAUG element occurs in the context of a putative G-quadruplex.

**FIG 4:**
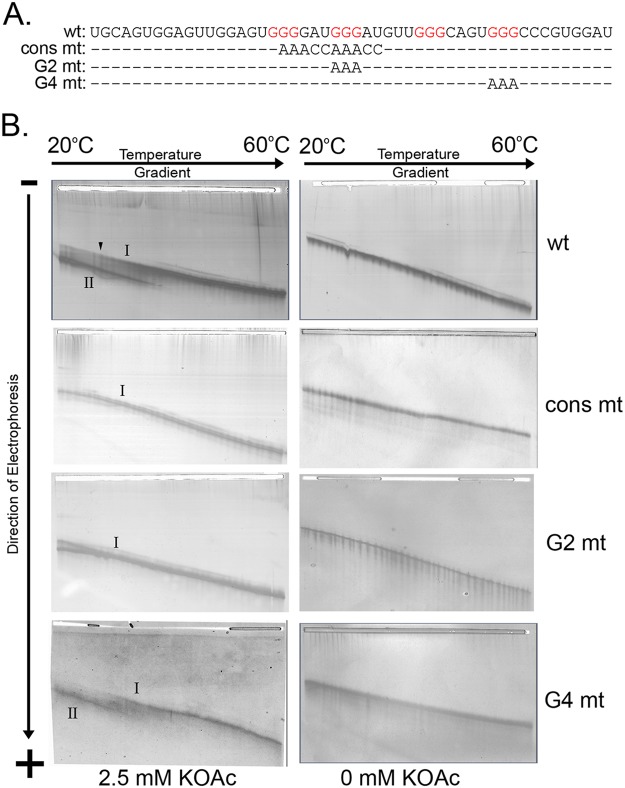
The 3′ UTR sequence containing the GGAUG element forms a structure consistent with a G-quadruplex. (A) RNA oligonucleotides used in the TGGE experiments highlighting differences with the wild-type oligonucleotide (top). (B) Silver-stained TGGE gel images in the presence (left) or absence (right) of 2.5 mM potassium acetate (KOAc). The linear RNA species is denoted as band I, and the faster-migrating species is denoted as band II. The oligonucleotide used in the analysis is indicated on the right.

Mammalian CNBP binds G-rich RNA sequences within putative G-quadruplex-forming mRNAs (13, 14, 22). To determine whether the secondary structure of the *RPL2* 3′ UTR is consistent with the formation of a G-quadruplex, we employed temperature gradient gel electrophoresis (TGGE) (23). G-quadruplex structures form spontaneously in the presence of potassium ions, and removal of the ion abrogates quadruplex formation (24, 25). As observed by TGGE, the wild-type (wt) 50-mer RNA containing the GGAUG element and four G-triplets (Fig. 4A) forms two types of structures in the presence of 2.5 mM potassium ions (Fig. 4B, top left). Two minor species with very similar migration behaviors at low temperature of the gel merge into a single conformation (band I) at ~38°C with lower mobility across the gradient, while a faster-migrating, major species (band II) corresponding to a more compact conformation is also more stable: in a broad, irreversible transition, this dominant structure denatures at approximately 38°C, comigrating with the slower species in band I at higher temperatures. This confirmation is dependent on the presence of potassium, it was not observed when potassium was omitted from the buffers (Fig. 4B, top right). Based on calculations using mFold (data not shown), it is reasonable to assume that the two minor species joining in band I with lower mobility represent linear RNAs containing one or two hairpins able to form independently along the sequence, while the faster-migrating species in band II is indicative of G-quadruplex formation in the presence of potassium ions, leading to a more compact shape with higher mobility.

To validate the presence of a Q-quadruplex structure under these conditions, we performed TGGE on mutant RNA oligonucleotides designed to disrupt potential Q-quadruplex formation. In these constructs, the consensus sequence was mutated (cons mt) abolishing the first two G-triplets, the second G-triplet was mutated (G2 mt), or the fourth G-triplet was mutated (G4 mt) (Fig. 4A). As demonstrated in the resulting TGGE panels of Fig. 4B, deletion of the consensus, which contains the G2 motif, abrogates G-quadruplex formation, as does mutation of the G2 sequence alone, allowing these RNA constructs to form only weak, hairpin-containing secondary structures similar to those seen in the wild type as minor band I-type species. Mutation of G4 leads to a weakening of the structure, as seen by a 2.5°C reduction (left shift) of the transition temperature in the gradient at which the structure unfolds to 31.4°C. It is possible that a less stable quadruplex may form with the one of the remaining G-doublets substituting for the G4 G-triplet in the structure, as this weakened structure retains potassium dependence for formation.

**FIG 5:**
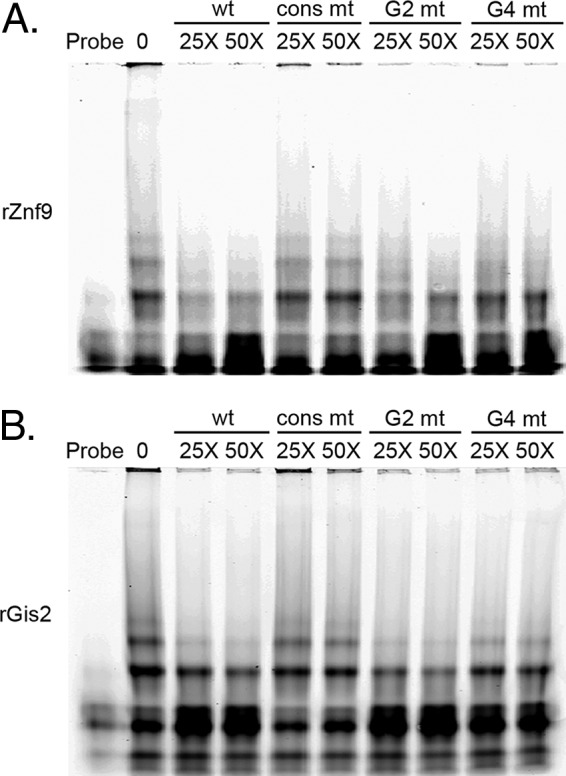
Oligonucleotides that contain the element but do not form structure still compete for binding. UV-cross-linked EMSA analysis with either recombinant Znf9 (A) or Gis2 (B) using the TYE705-labeled RNA oligonucleotide alone or with the indicated competitor oligonucleotide at 25× and 50× molar excess.

We then went on to investigate the role of G-quadruplex formation in protein binding. The same mutant oligonucleotides used in the TGGE experiments were used as competitor oligonucleotides in EMSAs with recombinant Gis2 and Znf9 (Fig. 5). Mutation of the binding element, as presented earlier, resulted in a loss of competition in the EMSAs with both Gis2 and Znf9. Despite differences in structure formation, only the consensus mutant lacked the ability to compete for protein binding with both Gis2 and Znf9, suggesting that the sequence of the element impacts binding more strongly than structure. Surprisingly, the G4 mutant exhibited weakened competition with Gis2, which may result from either differences in the primary sequence or aberrant structure formation that impacts accessibility to the binding element.

### Gis2 and Znf9 regulate basal RP transcript stability and translation.

**FIG 6:**
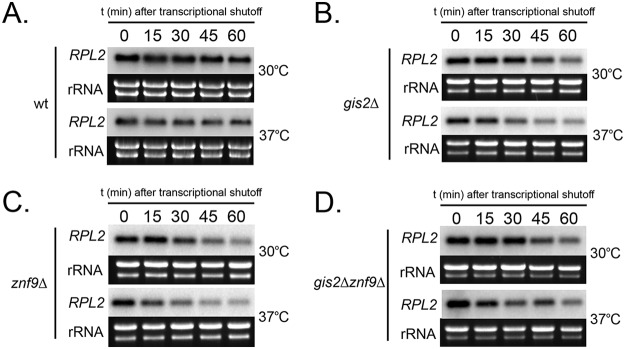
Acceleration of *RPL2* decay in response to temperature stress is unchanged by deletion of *GIS2*, *ZNF9*, or both genes. Analysis of RNA stability of *RPL2* by Northern blot analysis in a time course experiment following the addition of 1,10-phenanthroline to halt transcription. Prior to membrane transfer, separated rRNA bands were visualized using SYBR safe nucleic acid gel stain, and the resulting intensity was used as a total RNA loading control. (A to D) The wild type (A), *gis2*Δ mutant (B), *znf9*Δ mutant (C), and the *gis2*Δ *znf9*Δ double mutant (D) were grown to mid-log phase at 30°C or shifted to prewarmed 37°C medium.

RP transcripts are subject to accelerated mRNA decay during temperature stress in C. neoformans (2). Our initial intention was to identify the RNA binding proteins that mediate this acceleration in C. neoformans, and so we assessed the decay kinetics of *RPL2* in the wt, in the *gis2*Δ and *znf9*Δ single deletion mutants, and in the *gis2*Δ *znf9*Δ double mutant. In response to temperature stress, all strains exhibited an acceleration in *RPL2* decay rate, suggesting that neither Gis2 nor Znf9 is responsible for stress-responsive acceleration of *RPL2* decay (Fig. 6A to D).

**FIG 7:**
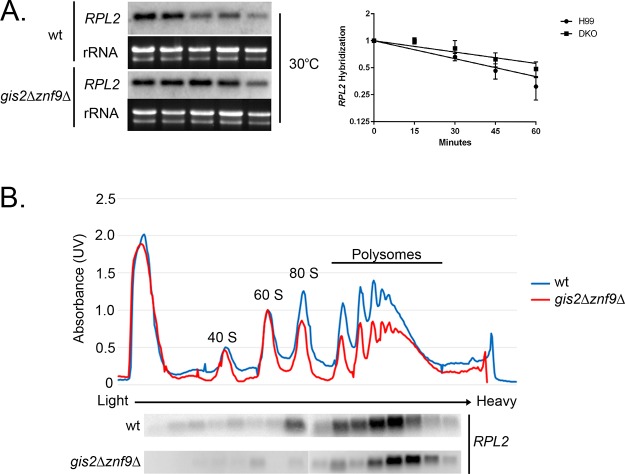
Deletion of *GIS2* and *ZNF9* affects basal RPL2 decay and polysome association. (A) Analysis of RNA stability at 30°C between the wild type and *znf9*Δ *gis2*Δ double mutant. RNA was isolated in a time course experiment after the addition of 1,10-phenanthroline to halt transcription. DKO, double knockout. (B) Analysis of polysome association of the *RPL2* mRNA in the wild type or *znf9*Δ *gis2*Δ double mutant during mid-log growth at 30°C. Fractions were collected from the polysome profile gradients, and RNA was isolated and subjected to Northern blotting for *RPL2*.

Our analysis of mRNA stability did reveal that the half-life of *RPL2* under unstressed conditions was longer in the *gis2*Δ *znf9*Δ double mutant than in the wild type, as the data lie on separate regression lines, suggesting that these proteins are affecting basal mRNA decay rates of RP transcripts (Fig. 7A). This is consistent with previous data from S. cerevisiae which demonstrated an increase in steady-state levels of RP transcripts in a *gis2*Δ mutant. To determine whether the increased stability impacted translation, we compared the polysome association of *RPL2* between the wild type and *gis2*Δ *znf9*Δ double mutant under unstressed conditions. As demonstrated in Fig. 7B, the *RPL2* mRNA is associated with higher-molecular-weight polysome fractions in the *gis2*Δ *znf9*Δ double mutant than in the wild type. This suggests that these proteins play a role in modulating translation of RP transcripts and that the modest increase in stabilization may be related to increased ribosome occupancy.

### Gis2 and Znf9 regulate resistance to stress.

**FIG 8:**
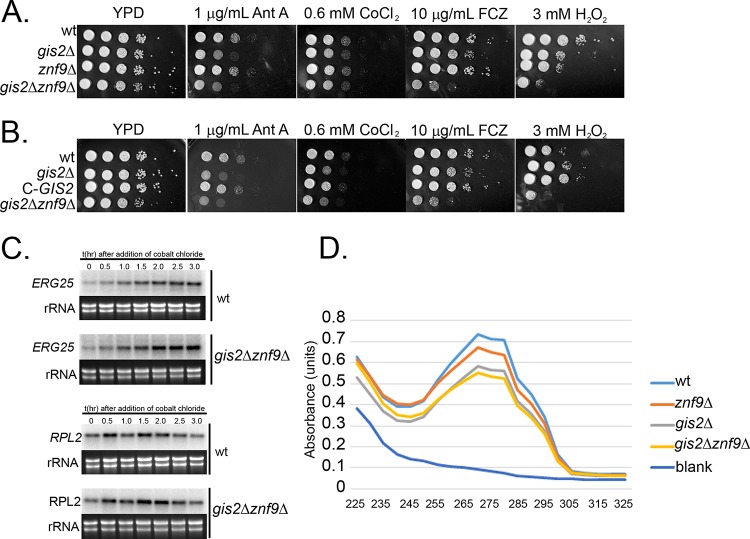
*GIS2* and *ZNF9* are required for stress resistance and ergosterol biosynthesis. (A) Spot plate analysis of the wild type, *gis2*Δ mutant, *znf9*Δ mutant, and *gis2*Δ *znf9*Δ double mutant on YPD medium alone or with the indicated stressor. (B) Spot plate assay of the wild type, *gis2*Δ mutant, complemented *gis2*Δ mutant, and *gis2* Δ*znf9*Δ double mutant on YPD alone or with the indicated stressor. The stressors in panels A and B are antimycin A (Ant A), cobalt chloride (CoCl_2_), fluconazole (FCZ), and peroxide (H_2_O_2_). (C) Analysis of *ERG25* and *RPL2* expression in response to cobalt chloride treatment in the wild type or *gis2*Δ *znf9*Δ double mutant. (D) Analysis of sterol content by heptane extraction in the wild type, *gis2*Δ mutant, *znf9*Δ mutant, and *gis2*Δ *znf9*Δ double mutant. Approximately 0.255 g (wet weight) (±0.005 g) of pelleted yeast cells were processed for sterol extraction for each strain. Data are representative of three biological replicates.

Human CNBP/Znf9 has been implicated in the regulation of translation, and the S. cerevisiae Gis2p interacts with the ribosome and is shuttled to stress granules under conditions of cellular stress (5, 13, 26). To determine whether there is a role for the C. neoformans CNBP homologues in stress tolerance, we assessed the sensitivity of the single and double mutants to a panel of stressors using spot plate analysis. As demonstrated in Fig. 8A, the *znf9*Δ mutant exhibited wild-type resistance to all stresses tested, whereas the *gis2*Δ mutant exhibited sensitivity to reagents that generate reactive oxygen species (antimycin A and peroxide), fluconazole, and cobalt chloride, with the deletion of both *GIS2* and *ZNF9* resulting in a synergistic phenotype with both peroxide and fluconazole. To ensure that the phenotype exhibited by the *gis2*Δ strain was due to the intended mutation, we introduced the wild-type *GIS2* gene in *trans* and assessed the mutant for restoration of wild-type phenotype by spot plate analysis. As demonstrated in Fig. 8B, introduction of the wild-type *GIS2* gene into the *gis2*Δ mutant did restore wild-type sensitivity to all stressors tested. Because sensitivity to cobalt chloride and fluconazole has been associated with the response to hypoxia and sterol biosynthesis, we went on to investigate the transcriptional activation of *ERG25*, a gene that is induced by hypoxia in an Sre1-dependent manner (27). We chose *ERG25* because its overexpression is sufficient to suppress the fluconazole sensitivity of an *sre1*Δ mutant (28). In response to cobalt chloride treatment, *ERG25* transcriptional induction was similar to that of the wild type, suggesting that the defect is downstream of Sre1 activation (Fig. 8C). Because there are many potential protein effectors in the sterol biosynthesis pathway that could be impacted at the level of translation, we chose to compare global sterol levels in the wild type and *gis2*Δ *znf9*Δ mutant using UV scanning of heptane extracts as performed previously (Fig. 8D) (29, 30). Comparison of sterol content in the wild type and in single and double mutants did reveal a reduction in sterols in the *gis2*Δ mutant that was exacerbated in the double mutant. This suggests that in addition to regulating basal decay and polysome association of RP transcripts, Gis2 and Znf9 participate in RP transcript-independent functions to regulate sterol biosynthesis and the response to stress. Future studies will investigate the regulatory role of these two proteins in translational regulation during stress.

## DISCUSSION

Posttranscriptional regulation is a means by which eukaryotic cells can fine-tune gene expression without global changes in mRNA synthesis rates. This control of mRNA fate can be imprinted at multiple steps along the mRNA life cycle, from the initial protein-mRNA associations during transcription, deposition of exon-junction complexes during splicing, association of RNA binding proteins before and after export, and association of translation factors. mRNA fate can be controlled by the association of RNA binding protein to either primary sequence or by structural elements. In this study, we present evidence that Gis2 and Znf9 bind to a primary mRNA sequence that occurs within a secondary structure but that the formation of the structure itself is dispensable for binding, as mutant RNA oligonucleotides that do not form the structure retain the ability to compete for binding. A recent study in mammalian cells concludes that CNBP binding within G-quadruplex-forming mRNA serves to prevent structure formation (13). Indeed, a computational investigation of the pervasiveness of G-quadruplex-forming mRNA sequences throughout the tree of life revealed that in eukaryotes, G-quadruplexes are common, but they are rare in bacterial mRNAs (31). It was further determined that although these sequences were found to form stable quadruplex structures *in vitro*, they were not structured *in vivo*. However, expression of eukaryotic G-quadruplex-forming RNAs in E. coli were found to form *in vivo* (31). This suggests that eukaryotes have a mechanism to either prevent G-quadruplex formation, unfold established structure, or both. The data with CNBP in humans in conjunction with our work present here suggest that CNBP orthologues may be a component of this mechanism to prevent G-quadruplex folding (13).

Znf9 and Gis2 are orthologues of human CNBP/Znf9 and S. cerevisiae Gis2p, respectively. Despite the ancient genome duplication, S. cerevisiae has only one gene that encodes a single CNBP orthologue, suggesting that Gis2 and Znf9 have arisen by selection in C. neoformans. Interestingly, CNBP in humans is regulated by methylation, and the C. neoformans Znf9 possesses a putative methylation consensus sequence, though in a different protein location than the human counterpart (21).

The biological functions of this family of proteins are not defined clearly. Inactivation of CNBP in mice is lethal, and trinucleotide repeat expansions in the upstream region of the human gene lead to myotonic dystrophy type II, indicating a pivotal role for these proteins in cellular homeostasis (15, 16). Several RNA binding studies of these proteins have revealed interactions with GA/U-rich sequences (13, 22). Our work approached the identification of these proteins from the opposing perspective, by performing an open-ended approach to identify RNA binding proteins that interact with a *cis* element identified by motif elicitation. Our determination that Gis2 and Znf9 bind this GGAUG motif is consistent with published consensus sequences identified in studies identifying CNBP and Gis2p targets (22).

Loss of Gis2 and Znf9 shifts *RPL2* expression toward higher ribosome occupancy, which suggests that Gis2 and Znf9 may play a negative regulatory role in the ribosome association of *RPL2* and potentially all RP mRNAs that contain the cognate element. The mechanism by which Gis2 and Znf9 effect this translational regulation is still unclear. One obvious possibility is found in the sequence of the consensus element—the AUG codon. Because AUG codons are able to be recognized by ribosomes for initiation, it is possible that in the absence of Gis2 and Znf9 binding, ribosomes may attempt to reinitiate in the 3′ UTR, thus increasing ribosome density on the mRNA. Perhaps then, Gis2 and Znf9 binding serves to mask this AUG codon to prevent ribosome initiation in the 3′ UTR and to terminate translation so as to regulate ribosome biogenesis. Future work will utilize ribosome profiling to determine whether in the absence of Gis2 and Znf9, ribosome footprints encompass the GGAUG element in the 3′ UTR.

Another reported function of CNBP proteins is their ability to act to promote the translation of mRNAs that contain internal ribosome entry site (IRES) elements (32–35). IRES *trans*-acting factor (ITAF) proteins promote the initiation of translation from mRNAs containing IRES elements in a manner that bypasses the requirement for the mRNA cap-binding complex. Under conditions of cellular stress, cap-dependent translation is often inhibited through the phosphorylation of eukaryotic translation initiation factor subunit 2-α (eIF2-α) (36, 37). Under these conditions, global translation is repressed, and translation initiation is slowed and becomes more stringent. To bypass this repression of translation initiation, IRES elements in the 5′ UTRs of mRNAs can be utilized to initiate translation. IRES elements were discovered in viral mRNAs and serve to promote viral protein synthesis during infection, when cellular translation is largely inhibited (reviewed in reference 38). The existence of cellular IRES elements has been documented in higher eukaryotes, and computational work has identified putative IRES-regulated processes in fungi in which 5′-UTR features are conserved across species (39). These enriched groups include hexose transporters, heat shock proteins, proton antiporters, ABC transporters, and a family of serine-rich proteins that are induced by hypoxia. Further work to define the role of Gis2 and Znf9 in regulating sterol biosynthesis and the oxidative stress response will investigate the role of these proteins as potential ITAFs in C. neoformans.

## MATERIALS AND METHODS

### Strains and media.

The strain of Cryptococcus neoformans used in these studies is a derivative of H99O that retains full virulence and melanization. C. neoformans was cultivated on YPD (1% yeast extract, 2% peptone, 2% dextrose) agar unless otherwise indicated. For all time course experiments, starter cultures in 3 to 5 ml of YPD were inoculated from stock plates and grown for 16 to 18 h at 30°C and 250 rpm in 15-ml snap-cap tubes. Cultures (30 to 50 ml) in baffled, cotton-plugged, 250-ml Erlenmeyer flasks were inoculated from the starter cultures at an optical density at 600 nm (OD_600_) of between 0.1 and 0.2 and allowed to reach mid-log phase (OD_600_ between 0.6 and 0.7) at which time the indicated manipulation was initiated and time course samples were taken. RNA isolation and Northern blotting were performed as described previously (2, 17).

The *znf9*Δ mutant strain was constructed as described previously (40). Briefly, approximately 1 kb upstream of *ZNF9* was PCR amplified with XbaI and BglII sites using primers F-ZNF9upKO-XbaI (F stands for forward, up stands for upstream, and KO stands for knockout) and R-ZNF9upKO-BglII (R stands for reverse) (Table 1). Approximately 500 bp downstream of *ZNF9* were PCR amplified using primers F-ZNF9downKO-BglII (down stands for downstream) and R-ZNF9KO-XhoI. The nourseothricin resistance cassette was PCR amplified with BglII and MunI restriction sites using primers F-NAT-BglII and R-NAT-MunI (Table 1). PCR-amplified products were digested with respective enzymes and cloned into pBluescript linearized with XbaI and XhoI such that NAT was ligated between the upstream and downstream flanking sequences. The knockout construct was PCR amplified, purified, precipitated onto gold microcarriers, and transformed into wild-type H99 by biolistic transformation as previously described (41). Nourseothricin-resistant colonies were screened by PCR to identify clones in which homologous recombination displaced the *ZNF9* gene, and Northern blot analysis was used to verify loss of gene expression.

The same procedure was used to knock out the *GIS2* gene in both the wild-type background and to create the *gis2*Δ *znf9*Δ double knockout. Primers F-GIS2up-XbaI and R-GIS2up-BglII and primers F-GIS2down-MunI and R-GIS2down-XhoI (Table 1) were used to amplify the upstream and downstream sequences of *GIS2*, respectively. The G418 resistance cassette was amplified using F-NEO-BglII and R-NEO-MunI (Table 1). PCR-amplified products were digested with respective enzymes and cloned into pBluescript linearized with XbaI and XhoI such that NEO was ligated between the upstream and downstream flanking sequences. The knockout construct was PCR amplified, purified, precipitated onto gold microcarriers, and transformed into wild-type H99 and the *znf9*Δ mutant strains by biolistic transformation as previously described (41). G418-resistant colonies were screened by PCR to identify clones in which homologous recombination displaced the *ZNF9* gene, and Northern blot analysis was used to verify loss of gene expression.

### Identification of shared *cis* element.

The sequences of the 3′ untranslated regions (UTRs) of ribosomal protein (RP) genes, or 500 bp downstream of the stop codon for genes without an annotated 3′ UTR, were obtained from the C. neoformans var. grubii database (https://www.broadinstitute.org/fungal-genome-initiative/cryptococcus-neoformans-serotype-genome-project). The sequences were then uploaded to the Multiple EM for Motif Elicitation (MEME) program (http://alternate.meme-suite.org), and the algorithm was programed to identify shared elements of 3 to 15 bases in length (18, 19).

### Isolation of whole-cell lysate.

Whole-cell lysate from a mid-log culture of the wild type, H99, grown at 30°C and 250 rpm was obtained by mechanical disruption. Briefly, cells were pelleted by centrifugation at 4,000 rpm and washed with an equal volume of sterile deionized water. The pellet was transferred to a microcentrifuge tube and centrifuged at 14,000 rpm for 30 s, and the residual supernatant was aspirated. The pellet was resuspended in 0.10 volumes of lysis buffer (15 mM HEPES [pH 7.4], 10 mM KCl, 5 mM MgCl_2_, 10 µl/ml HALT protease inhibitor [Thermo Scientific]), and glass beads were added until the cell suspension was saturated with 2 mm of dry beads on top. After the cell suspension was incubated on ice for 10 min, the cells were lysed by five cycles of vortexing for 30 s followed by 30 s of incubation on ice. The supernatant was transferred to a new microcentrifuge tube on ice followed by centrifugation at 14,000 rpm and 4°C for 10 min. The cleared lysate was transferred to a new tube on ice.

### Protein capture assay.

One hundred micrograms of protein from H99 whole-cell lysate was transferred to a microcentrifuge tube. MgCl_2_ was added to a final concentration of 3 mM, tRNA was added to 0.1 mg/ml, and 10 µg of primer Biotin-RPL2-3′UTR or Biotin-RPL2-3′UTRmt (mt stands for mutant) (Table 1) was added. The reaction mixture was incubated at 4°C for 90 min with rotation. KCl was then added to a final concentration of 40 mM, heparin was added to 50 µg/ml, and the reaction mixture was incubated at 4°C with rotation overnight. The reactions were UV cross-linked for 15 min and then added to prewashed high-capacity NeutrAvidin agarose resin (Pierce) and incubated with rotation for 1 h. The resin was washed three times with binding buffer (3 mM MgCl_2_, 50 µg/ml heparin, 0.1 mg/ml tRNA) with increasing concentrations of KCl (250 mM. 500 mM, 1 M). The resin was washed one final time with binding buffer containing no KCl. Resin was boiled in SDS sample buffer at 95°C for 5 min, and the eluate was loaded onto a 6 to 12% polyacrylamide Bis/Tris gel and electrophoresed at 200 V. Protein bands were detected by silver staining. Bands of interest that appeared in reactions with the biotin-RPL2-3′UTR oligonucleotide but not with the biotin-RPL2-3′UTRmt oligonucleotide were excised and destained.

### LC-MS/MS protein identification.

Liquid chromatography coupled to tandem mass spectrometry (LC-MS/MS) was performed at the Seattle Biomedical Research Institute Proteomics Core Facility. Gel bands were digested with trypsin, desalted, and analyzed with an Orbitrap mass spectrometer. A database search was conducted using the C. neoformans var. grubii H99 protein database (Cryptococcus neoformans var. grubii H99 Sequencing Project, Broad Institute of Harvard and MIT [https://www.broadinstitute.org/]) after adding common contaminants. The cutoff for peptide identification (ID) was an error rate of ≤0.05, and for protein ID, a probability score of ≥0.9.

### Production and purification of recombinant proteins.

*GIS2* or *ZNF9* was amplified from cDNA with primers F-rGIS2-BglII and R-rGIS2-BglII (r stands for recombinant) for *GIS2* or F-GIS2cDNA-BamHI and R-GIS2cDNA-BamHI for *ZNF9* (Table 1). PCR products were digested with BglII (*GIS2*) or BamHI (*ZNF9*), ligated into BamHI-linearized pET14b vector in frame, and transformed into electrocompetent E. coli DH10B cells. Purified plasmid was then transformed into chemically competent *E*. *coli* BL21(DE3)pLysS cells by chemical transformation. Cells were grown to an OD_600_ of 0.6 in 100 ml of LB with antibiotics at 37°C and 250 rpm, and protein expression was induced with 0.1 mm isopropyl-β-d-thiogalactopyranoside (IPTG) for 2 h at 30°C. Cells were pelleted by centrifugation at 4,000 rpm and 4°C for 20 min followed by freezing. Pellets were thawed on ice and resuspended in 5 ml of lysis buffer (50 mM NaH_2_PO_4_, 300 mm NaCl, 10 mM imidazole [pH 8.0]) and 1 mg/ml lysozyme, followed by incubation on ice for 30 min. Cells were sonicated on ice and then pelleted at 10,000 × *g* and 4°C for 25 min. The cleared lysate was applied to a preequilibrated nickel-nitrilotriacetic acid (Ni-NTA) gravity flow column (Qiagen). The column was washed according to the manufacturer’s protocol, and protein was eluted twice with 3 ml of elution buffer. Elutions were dialyzed using the Slide-A-Lyzer system (Pierce) according to the manufacturer’s protocol with EMSA buffer for buffer exchange.

### Electrophoretic mobility shift assays.

All electrophoretic mobility shift assay (EMSA) reaction mixtures contained 0.5 pmol of the TYE705-labeled oligonucleotide probe (Table 1) and contained 4 µl of 5× EMSA buffer (75 mM HEPES [pH 7.4], 200 mM KCl, 25 mM MgCl_2_, 25% glycerol), and were brought to a final volume of 20 µl with sterile deionized water. For reactions with whole-cell lysate, 5 µg of protein was added. Reactions with recombinant protein contained 5 µg of purified recombinant protein. For competitive EMSA reactions, 25 pmol of either cold competitor or mt competitor oligonucleotide (Table 1) was added. For native EMSA, reaction mixtures were incubated at room temperature for 20 min, loaded onto a DNA retardation gel, and electrophoresed at 100 V. For cross-linked EMSAs, reaction mixtures were incubated at room temperature for 20 min, UV cross-linked on ice for 10 min, loaded onto 4 to 12% Bis-Tris SDS-polyacrylamide gels, and electrophoresed at 200 V. Gels were imaged using a LiCor Odyssey infrared imaging system.

### Preparation of RNA oligonucleotides for TGGE.

High-performance liquid chromatography (HPLC)-purified RNA oligonucleotides (IDT) used in these studies are indicated in Table 1. To gel purify, 10 µg of synthesized RNA was loaded into a 0.5× TBE (1× TBE is 89 mM Tris, 89 mM boric acid, and 10 mM EDTA) 1.5-mm acrylamide gel with 8 M urea. Constant voltage was applied at 60° via a circulating water bath (Fisher Owl electrophoretic apparatus). RNA in the gel was visualized using a thin-layer chromatography (TLC) plate exposed to UV light. The band corresponding to the intact 50-bp region was excised, frozen, crushed, and resuspended in RNA elution buffer (0.5 M ammonium acetate, 1 mM EDTA, 0.1% SDS). RNA was separated from extraction buffer using phenol-chloroform, precipitated using 2.5 volumes of ethanol, and resuspended in 50 µl of Tris-EDTA (TE) buffer.

### Temperature gradient gel electrophoresis.

Temperature gradient gel electrophoresis (TGGE) was performed as described previously (23, 42). Briefly, 500 ng of RNA was diluted in 375-µl total volume of deionized water with or without 2.5 mM potassium acetate. Samples were denatured on a 95° heat block for 10 min, flash frozen with liquid nitrogen, and defrosted on ice before loading on the TGG. TGGE was run on the Biometra TGGE Maxi System as described previously (43). Briefly, gels were 20 by 19 by 0.1 cm on film support (GelBond-PAG; GE/Amersham) and contained 12% (wt/vol) acrylamide, 0.17% bisacrylamide, 0.05% (vol/vol) *N*,*N*,*N*′,*N*′-tetramethylethylenediamine (TEMED), and 0.05% (wt/vol) ammonium peroxidisulfate for initiating the polymerization, and 0.2× TBE. Electrophoresis was performed in three steps. (i) Samples were applied to the 16- by 0.4-cm sample slot of the horizontally mounted, precooled gel, and RNA was allowed to migrate several millimeters into the matrix at a uniform temperature of 10°C and 400 V for 10 min. (ii) A constant temperature gradient was established in the gel from 20° to 60°C. (iii) Electrophoresis was resumed with the applied temperature gradient for 1.0 to 1.5 h at 400 V. RNA was detected by silver staining (44).

### Stability assays and Northern blot analyses.

Stability assays were conducted as previously described. Briefly, mid-log-phase cells grown at 30°C in YPD were either kept at 30°C or pelleted and resuspended in prewarmed 37°C YPD. Transcription was inhibited by the addition of 1,10-phenanthroline (250 µg/ml), and cultures were returned to incubation at respective temperatures. Aliquots were pelleted every 15 min for 1 h. Cells were lysed by mechanical disruption with glass beads, and RNA was extracted using RNeasy column purification (Qiagen). For each sample, 3 µg of RNA was denatured, electrophoretically separated through 1% agarose–formaldehyde gel, and transferred to a nylon membrane. The membrane was UV cross-linked, hybridized with a ^32^P-labeled *RPL2* probe (2), and imaged by phosphorimaging. Hybridized *RPL2* signal was normalized to rRNA gel bands. The half-life of *RPL2* was determined by nonlinear regression of normalized *RPL2* over time (Graphpad).

### Polysome profiling.

Strains were inoculated at a density of OD_600_ of 0.15 in 200-ml total volume from an overnight starter culture. Cells were grown in a 2-liter baffled flask with shaking at 250 rpm and 30°C for 5 to 6 h, reaching an OD_600_ of ~0.55 to 0.65. Polysome profiles were obtained as described previously (1). Yeast cells were then harvested in the presence of 0.1 mg/ml cycloheximide (Acros) and pelleted immediately at 4,000 rpm for 2 min at 4°C. The yeast pellet was then flash frozen in liquid nitrogen, resuspended, and washed in polysome lysis buffer (20 mM Tris-HCl [pH 8], 2.5 mM MgCl, 200 mM KCl, 1 mg/ml heparin [Sigma], 1% Triton X-100, 0.1 mg/ml cycloheximide). Yeast cells were then lysed mechanically by glass bead disruption, resuspended in 500 µl of polysomal lysis buffer, and centrifuged for 10 min at 16,000 × *g* and 4°C to obtain the cytosolic portion of the lysate. Total RNA (250 µg) in a 250-µl total volume was layered on top of the polysome sucrose gradient (10% to 50% linear sucrose gradient, 20 mM Tris-HCl [pH 8], 2.5 mM MgCl, 200 mM KCl, 1 mg/ml heparin, 0.1 mg/ml cycloheximide). Gradients were subjected to ultracentrifugation at 39,000 rpm in an SW-41 rotor at 4°C for 2 h. Following centrifugation, sucrose gradients were pushed through a flow cell using a peristaltic pump, and RNA absorbance was recorded using Teledyne’s UA-6 UV-visible (UV-Vis) detector set at 254 nm. Absorption output was recorded using an external data acquisition device (DataQ). Fractions were then collected following absorption using a Teledyne retriever 500 set to collect 16-drop fractions.

To extract RNA, fractions were suspended in 3 volumes of 100% ethanol and incubated at −80°C for 12 to 16 h. Precipitate was collected via centrifugation at 16,000 × *g* at 4°C for 20 min and resuspended in 250 µl warm RNase-free water with the addition of Trizol LS (Invitrogen). RNA was extracted per the manufacturer’s instruction. Purified RNA was resuspended in 30 µl RNase-free water. A third of this volume was used in subsequent Northern blot analyses.

### Ergosterol scan analysis.

Sterol levels were measured as described previously with the following alterations (29, 30). Strains were inoculated at a density of OD_600_ of 0.15 in 50 ml from an overnight starter culture. Cells were grown in a shaking incubator at 30°C and 250 rpm for 24 h. The cells were harvested and washed twice with water. Approximately 0.25 g (wet weight) (±0.005 g) of pelleted yeast cells was suspended to 300 µl in 20% KOH and 60% ethanol and incubated at 85°C for 1 h. Dissolved culture was allowed to return to room temperature before the addition of 100 µl of water and 300 µl of heptane. After 3 min of vortexing, the heptane layer was removed, and the extracted sterols were diluted 1:2 with ethanol. Diluted samples were placed in a quartz cuvette and measured spectrophotometrically from 225 to 325 nm on a SpectraMax M5 plate reader/spectrophotometer (Molecular Devices).

### Accession number(s).

Gene loci (GenBank accession numbers) for the genes used in this study are as follows: *ZNF9* (CNAG_01273), *GIS2* (CNAG_02338), *ERG25* (CNAG_01737), and *RPL2* (CNAG_05232).

### Data availability.

Deletion strains produced in this study are available upon request. No large-scale bioinformatic data were produced in this study.
